# Watt's Letter on an Aneurism Needle

**Published:** 1811-03

**Authors:** John James Watt

**Affiliations:** Bartholomew Close, 94


*To the Editors of the Medical and Physical Journal.*

Gentlemen,

In the Number of your Journal for January last, a letter is inserted, in which an attempt has been made by Mr. H. Earle, to deprive me of the invention of an Aneurismal Needle described under my name. As it is my intention, however, not to surrender the claim I hold to the originality of this contrivance, *which is well known at St. Bartholomew's Hospital to be my own*, I must beg the favour of your allowing me a place in your useful Journal, merely to shew, that I have not condescended to become the copyist of that gentleman.

I am, Gentlemen,
Yours, most respectfully,
JOHN JAMES WATT.

*Bartholomew Close*, 94,

*Feb.* 14, 1811.

